# Broadband coherent Raman spectroscopy running at 24,000 spectra per second

**DOI:** 10.1038/srep21036

**Published:** 2016-02-15

**Authors:** Kazuki Hashimoto, Megumi Takahashi, Takuro Ideguchi, Keisuke Goda

**Affiliations:** 1 Department of Chemistry, University of Tokyo–7-3-1 Hongo, Bunkyo-ku, Tokyo 113-0033, Japan; 2 Research Centre for Spectrochemistry, University of Tokyo–7-3-1 Hongo, Bunkyo-ku, Tokyo 113-0033, Japan; 3 Department of Electrical Engineering, University of California, Los Angeles–Los Angeles, CA 90095, USA; 4 Japan Science and Technology Agency–7 Gobancho, Chiyoda-ku, Tokyo 102-0076, Japan

## Abstract

We present a Fourier-transform coherent anti-Stokes Raman scattering (FT-CARS) spectroscopy technique that achieves broadband CARS measurements at an ultrahigh scan rate of more than 20,000 spectra/s – more than 20 times higher than that of previous broadband coherent Raman scattering spectroscopy techniques. This is made possible by an integration of a FT-CARS system and a rapid-scanning retro-reflective optical path length scanner. To demonstrate the technique’s strength, we use it to perform broadband CARS spectroscopy of the transient mixing dynamics of toluene and benzene in the fingerprint region (200–1500 cm^−1^) with spectral resolution of 10 cm^−1^ at a record high scan rate of 24,000 spectra/s. Our rapid-scanning FT-CARS technique holds great promise for studying chemical dynamics and wide-field label-free biomedical imaging.

Spectroscopy based on coherent Raman scattering (CRS) including coherent anti-Stokes Raman scattering (CARS) and stimulated Raman scattering (SRS) is a method widely used in scientific research^1^,^2^,^3^,^4^. CRS spectroscopy has been employed in a label-free manner to identify vibrational signatures of molecules in diverse biomedical applications such as cancer detection^5^, drug delivery^6^, endoscopy^7^,^8^, single molecule analysis^9^, and lipid metabolism^10^,^11^. In many applications including these, the ability to acquire the CRS signal at high scan rates is critical for multi-dimensional blur-free imaging of moving tissues and fast transient dynamics^4^. It is also important for high-throughput applications that require scanning a large tissue area or screening a large population of cells in a short period of time.

To exploit diverse applications of CRS spectroscopy in practical settings, enormous efforts have been made toward high CRS spectrum acquisition rate over the last decade^12^,^13^,^14^,^15^,^16^,^17^,^18^,^19^,^20^,^21^. High-speed CRS operation with a single spectral element has been demonstrated for video-rate CRS microscopy^12^,^13^. Also, to fully utilize the potential of CRS spectroscopy, broadband spectral acquisition at high scan rate has been realized by using multichannel detection^14^,^15^,^16^,^17^ or frequency-swept lasers^18^,^19^,^20^,^21^. To the best of our knowledge, the highest spectrum acquisition rate is reported to be about 1,000 spectra/s over a broadband spectrum of ~1000 cm^−1^ (ref. 14).

In this Letter, we present a CARS technique that achieves broadband CARS measurements at an ultrahigh scan rate of more than 20,000 spectra/s – more than 20 times higher than that of previous broadband CRS spectroscopy techniques^4^,^14^,^15^. This is enabled by an integration of a rapid-scanning retro-reflective optical path length scanner into Fourier-transform CARS (FT-CARS)^22^,^23^,^24^,^25^,^26^,^27^,^28^. As a proof-of-concept demonstration, we demonstrate ultrafast CARS spectroscopy in the fingerprint region (200–1500 cm^−1^) with spectral resolution of 10 cm^−1^ at a record high scan rate of 24,000 spectra/s. Furthermore, we use the technique to observe the transient dynamical process of mixing toluene and benzene. This ultrafast FT-CARS technique is expected to be valuable for studying chemical dynamics and wide-field label-free biomedical imaging in which high spectrum acquisition rates are required.

FT-CARS spectroscopy is a version of time-domain coherent Raman scattering spectroscopy whose principle is analogous to impulsive stimulated Raman scattering^29^. In FT-CARS, a train of dual pulses with a time delay with respect to each other is used to excite and probe the target molecular vibrations. The first pulse excites the vibrations that periods are longer than the pulse width, which is then probed by the second pulse. The time delay is scanned by every pulse pair to pulse pair, which generates anti-Stokes or Stokes pulses alternately. When the probe pulse probes the molecular vibration out-of-phase to the vibration, it gains energy from the molecules (anti-Stokes shift). The resulting filtered anti-Stokes signal is encoded in the time-domain interferogram, which is detected by a single-pixel photodetector. The CARS spectrum can be obtained by taking the Fourier-transform of the interferogram.

Our rapid-scanning FT-CARS system is schematically shown in Fig. 1 (see Methods for details). The optical source is a transform-limited Ti:Sapphire femtosecond pulse laser with a center wavelength of 792 nm, a pulse width of 17 fs, and a pulse repetition rate of 75 MHz. A pulse from the laser is first sent into a Michelson interferometer in which the pulse is split by its polarizing beamsplitter (PBS). In one of the interferometer arms (scanning arm), the split pulse is directed toward a rapid-scanning retro-reflective optical path length scanner whose design is analogous to the scanning delay line reported in ref 30. The path length scanner consists of 12-kHz resonant scanning mirror, a 1-inch concave mirror (f = 50 mm), and a rectangular mirror in a retro-reflective 4 f configuration such that the returned pulse from the path length scanner travels back along the same path as the incident pulse except for the time delay produced by the scanner. At the same PBS, the returned pulse recombines with a time delay with the other split pulse which returns from the other arm of the interferometer (reference arm), resulting in a train of dual collinear pulses that can be variably time-delayed with respect to each other. The dispersion of the whole system is compensated by two pairs of chirped mirrors. The dual-pulse train is focused onto a sample after a long-pass filter with a cut-off wavelength of 750 nm to induce the third-order nonlinear interaction in the sample. The generated anti-Stokes signal is detected by an avalanche photodetector after removing the remaining excitation light with a short-pass filter with a cut-off wavelength of 738 nm. After an electrical low-pass filter for removing fundamental beam repetition frequency, the photodetector signal is sampled by an externally clocked digitizer (See the Methods for details). This measurement can be repeated with trains of dual collinear pulses with an increasing time delay with respect to each other in order to obtain the interferogram in which the CARS signal is encoded. The CARS spectrum can be obtained by taking the Fourier-transform of the interferogram. The acquisition of the CARS spectrum can be repeated at the rate equivalent to the scan rate of the path length scanner multiplied by a factor of two (from the round trip of the scanner). Here the path length scanner produces a path length difference of up to 1 mm between the two interferometer arms (corresponding to a CARS spectral resolution of 10 cm^−1^) at a scan rate of 24,000 scans/s (corresponding to 41.7 μs/scan). The CARS spectral range is estimated to be from 200 cm^−1^ (limited by the optical filtering) to 1500 cm^−1^ (limited by the pulse width at the sample).

We first characterized the performance of the rapid-scanning FT-CARS system for broadband CARS measurements. Figure 2a shows a series of continuous-wave interferograms produced by the rapid-scanning retro-reflective optical path length scanner using a continuous-wave laser at 1064 nm. Figure 2b shows the time-varying path length difference between the interferometer arms. It clearly shows nonlinearity caused by the nonlinear motion of the rapid-scanning retro-reflective optical path length scanner. We utilized the continuous-wave interferograms as an external clock for the digitizer to linearize the CARS interferograms.

Figure 3 shows a continuous series of CARS interferograms obtained by the continuously running FT-CARS system. Here the sample is liquid toluene in a cuvette exposed to an irradiation of 130 mW of average power. The large peaks that appear every 83.3 μs (corresponding to two CARS scans or one oscillation of the scanner) are caused by the non-resonant background due to the zero time delay between the interferometer arms and are removed to avoid spectral distortions. The inset shows a zoom of the temporal waveform in which the CARS interferogram is encoded as a modulation to the waveform.

We next obtained and plotted in Fig. 4 a time-sequenced series of the CARS spectra by segmenting and Fourier-transforming the interferograms from the continuous time-domain waveform shown in Fig. 3. In the process of the Fourier transformation, we removed the large peaks in Fig. 3 (which appear every 83.3 μs) caused by the non-resonant background due to the zero time delay between the interferometer arms in order to avoid unwanted spectral distortions. We also applied a triangular apodization to the CARS spectra. Figure 4 clearly indicates our technique’s ability to perform CARS spectroscopy in the fingerprint region at an ultrahigh scan rate of 24,000 spectra/s. The characteristic lines of toluene (large peaks at 786, 1003, and 1030 cm^−1^ and small peaks at 520 and 1216 cm^−1^) can be seen in the spectra. Here the spectral resolution is as high as 10 cm^−1^ (unapodized) or 18 cm^−1^ (apodized). The signal-to-noise ratio of our method was measured to be 34 at 786 cm^−1^. As with conventional FT-CARS, our method has a non-uniform CARS intensity distribution over the measurable spectral region (200–1500 cm^−1^). Signal-to-noise ratio is reduced at the edges of the spectrum due to the lower excitation efficiency of impulsive stimulated Raman scattering at higher wavenumbers and the lower signal collection efficiency at lower wavenumbers caused by the optical filter for the CARS signal detection. The long-term spectral jittering of the method for a duration of 41.7 ms (n = 1000 in Fig. 3) was also measured to be less than the spectral resolution (10 cm^−1^), meaning that the method has high stability.

We then evaluated the dependence of the CARS signal on the concentration of toluene by measuring the peak CARS signal intensity at 786 cm^−1^ at several different concentrations of toluene. Figure 5 clearly shows that the CARS signal intensity is proportional to the concentration of toluene. This linear dependence of the CARS signal on the concentration of toluene is due to our heterodyne detection of the signal with a local oscillator derived from the strong non-resonant background^31^. From the noise level, the detection limit of our system (evaluated at the toluene peak at 786 cm^−1^) on the concentration of toluene is found to be 3%.

In order to demonstrate the rapid-scanning FT-CARS system’s capability of probing transient chemical dynamics, we used it to observe the mixing process of toluene and benzene (both in liquid state) at the same scan rate of 24,000 spectra/s. Here we added benzene to toluene in a cuvette and mixed them. The evolution of the CARS spectrum of this sample during the mixing process is shown in Fig. 6a. In the beginning (before the mixture), only the CARS signal of toluene is present. Over time, the CARS signatures of the two species are present, indicating that they are mixed at the focal point of the incident light. Figure 6b shows the temporal variation in the peak intensity of the CARS signal at 786 cm^−1^ and 990 cm^−1^, corresponding to toluene and benzene, respectively. The alternating variations in the toluene and benzene concentrations verifies the mixing dynamics of two liquid chemicals with a temporal resolution of 41.7 μs. This demonstration firmly shows that our technique is an effective tool for studying fluidic mixing – an important area of research for the characterization and optimization of micro- and nano-fluidic devices in food, chemical, and pharmaceutical industries^32^,^33^.

In conclusion, we have demonstrated a FT-CARS technique that enables broadband CARS spectroscopy (200–1500 cm^−1^) with high spectral resolution of 10 cm^−1^ at a record high scan rate of 24,000 spectra/s – more than 20 times higher than that of previously reported methods. This technique is based on a combination of a FT-CARS system and a rapid-scanning retro-reflective optical path length scanner with a large scan range of 1 mm and a high scan rate of 12 kHz. In addition, we have shown its application to probing the mixing dynamics of two chemical species as an effective tool for studying micro- and nano-fluidic devices. The technique can further be improved for better performance or broader utility by making simple modifications to its setup. First, the technique can be integrated into a 2D lateral scanning setup with a pair of galvanometric scanners or a 2D piezoelectric stage for 2D CARS imaging in the fingerprint region. Second, if the path length scanner is implemented in both arms of the Michelson interferometer with a phase difference of π, the time delay between the two arms can be doubled, leading to a factor of two higher spectral resolution. Finally, employing a few-cycle pulse Ti:Sapphire laser can expand its spectral bandwidth up to 4800 cm^−1^ (ref. 26). Our rapid-scanning FT-CARS technique holds great promise for studying chemical dynamics and wide-field label-free biomedical imaging in food, chemical, and pharmaceutical industries as well as medicine.

## Methods

### Design of the rapid-scanning FT-CARS system

The optical source of our FT-CARS system is a transform-limited Ti:Sapphire femtosecond pulse laser (SynergyTM, Femtolasers) with a center wavelength of 792 nm, a bandwidth of 47 nm, a pulse width of 17 fs, and a pulse repetition rate of 75 MHz. The laser beam is linearly polarized by a polarizer with high extinction ratio (PLC-10-800, OptoSigma) and is split into two orthogonally polarized beams by the PBS (PBSW-10-800, OptoSigma) after passing it through an achromatic half-wave plate (AHWP05M-980, Thorlabs) with which the power ratio between the two split beams can be optimized. One beam is directed toward the optical path length scanner in one of the Michelson interferometer arms while the other is directed toward a fixed mirror in the other interferometer arm. The polarization of each returned beam is rotated by 90 degrees by an achromatic quarter-wave plate (QWP) (AQWP05M-980, Thorlabs) in each arm of the interferometer. The two returned beams are recombined at the same PBS. The combination of the PBS and QWP acts as an isolator that prevents the beam from travelling back to the laser. The optical path length scanner reflects the pulse along the same path as the incident pulse except for the time delay. The group-velocity dispersion of all the dispersive elements in the FT-CARS system is compensated by two pairs of chirped mirrors by a total amount of −2260 fs^2^ (−175 fs^2^/reflection × 12 reflections and −40 fs^2^/reflection × 4 reflections). The dual pulse train is focused onto a sample in a 2-mm-thick cuvette by an aspherical lens (C240TME-B, NA = 0.5, Thorlabs) to induce the third-order nonlinear interaction. The scattered beam (anti-Stokes scattering signal) is collected by another aspherical lens (C240TME-B, NA = 0.5, Thorlabs) and detected by an avalanche photodetector (APD120A/M, Thorlabs). To efficiently reduce the noise, the excitation light is rejected by a combination of a long-pass filter (FELH0750, OD > 5, Thorlabs) with a cut-off wavelength of 750 nm and a short-pass filter (FESH0750, OD > 5, Thorlabs) with a cut-off wavelength of 738 nm before and after the sample, respectively. The cut-off wavelength of the filters were optimized by slightly tilting them against the beam.

### Design of the optical path length scanner

The design of the rapid-scanning optical path length scanner is similar to the one used for optical coherence tomography in Ref. 30. In our system, the path length scanner consists of an elliptically shaped 12-kHz resonant scanning mirror with major and minor radii of 6 mm and 4 mm, respectively (CRS 12 kHz, Cambridge Technology), an 1-inch concave mirror (f = 50 mm), and a 25 × 36 mm rectangular mirror in a retro-reflective 4 f configuration such that the returned pulse from the path length scanner travels back along the same path as the incident pulse except for the time delay produced by the scanner. The path length scanner produces a path length difference of up to 1 mm (due to its optical scan angle of 10 degrees) between the two interferometer arms (corresponding to a CARS spectral resolution of 10 cm^−1^).

### Sampling of the CARS signal

The detector signal is filtered with an electric low-pass filter (BLP-30+, Mini-Circuits) and sampled by a digitizer (ATS9440, Alzartech) with the external clock. The external clock is provided by the interference of a 1064-nm continuous-wave laser (QLD1061, QDLaser) in the Michelson interferometer. The beam of the continuous-wave laser is spatially overlapped with that of the pulsed laser with a dichroic beamsplitter (DMLP950, Thorlabs). It is separated by another dichroic beamsplitter after the interferometer and detected by a photodiode (PDA10CF-EC, Thorlabs). The ac part of the detector signal is extracted by a bias-tee (ZFBT-4R2GW+, Mini-Circuits) and used as an external clock of the digitizer.

### Procedure of the mixing experiment

The cuvette was first filled with toluene. Shortly after the digitizer started data acquisition (roughly a second after), benzene was dropped into the cuvette by a glass pipet. The mixing process was measured for a few seconds.

## Additional Information

**How to cite this article**: Hashimoto, K. *et al.* Broadband coherent Raman spectroscopy running at 24,000 spectra per second. *Sci. Rep.* **6**, 21036; doi: 10.1038/srep21036 (2016).

## Figures and Tables

**Figure 1:**
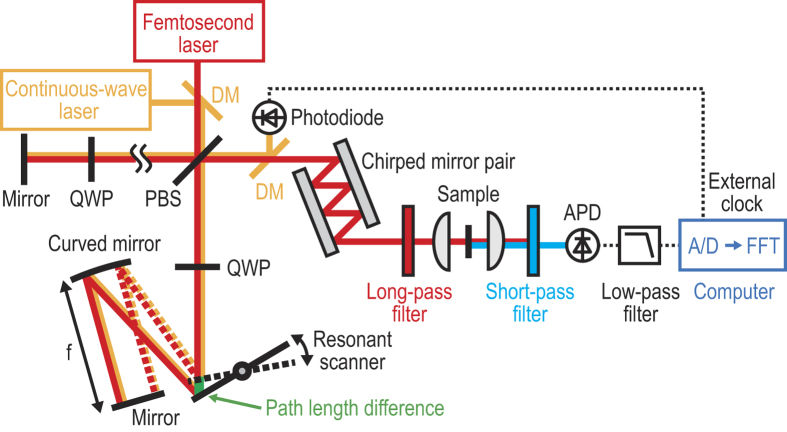
Schematic of the rapid-scanning FT-CARS system. A pulse from the laser is first sent into the Michelson interferometer with a rapid-scanning retro-reflective optical path length scanner in one of its arms. The scanner is arranged in such a way that the returned pulse from the scanner travels back along the same path as the incident pulse except for the time delay produced by the scanner. A train of dual collinear pulses with a variable time delay with respect to each other exits the interferometer and focused onto the sample. The resulting filtered anti-Stokes signal is detected by the avalanche photodetector and digitized by the digitizer with an external clock from the continuous-wave interferograms. This measurement can be repeated with trains of dual collinear pulses with an increasing time delay with respect to each other in order to obtain the interferogram in which the CARS signal is encoded. The CARS spectrum can be obtained by taking the Fourier transform of the interferogram. The acquisition of the CARS spectrum can be repeated at the rate equivalent to the scan rate of the path length scanner multiplied by a factor of two (from the round trip of the scanner). DM: dichroic mirror, PBS: polarizing beamsplitter, QWP: quarter-wave plate, APD: avalanche photodetector.

**Figure 2:**
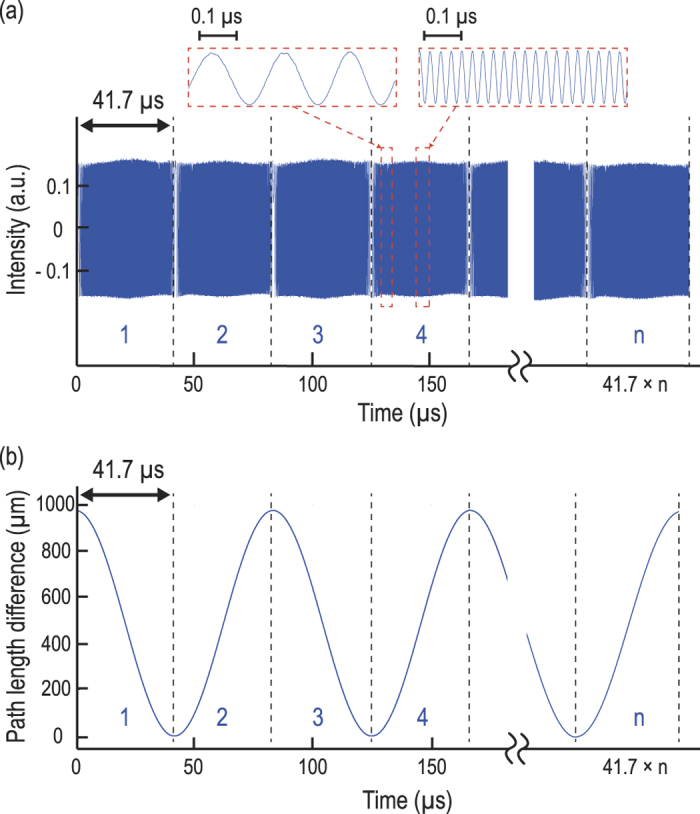
Characterization of the Michelson interferometer with the path length scanner. (**a**) Series of continuous-wave interferograms produced by the rapid-scanning retro-reflective optical path length scanner. The insets show enlarged views of interferograms. (**b**) Time-varying path length difference between the interferometer arms produced by the rapid-scanning retro-reflective optical path length scanner. The path length difference is obtained by enumerating the zero-crossing points in one of the continuous-wave interferograms shown in Fig. 2a. The nonlinear relation between the path length difference and time is corrected by an external clock timed by the zero-crossings.

**Figure 3:**
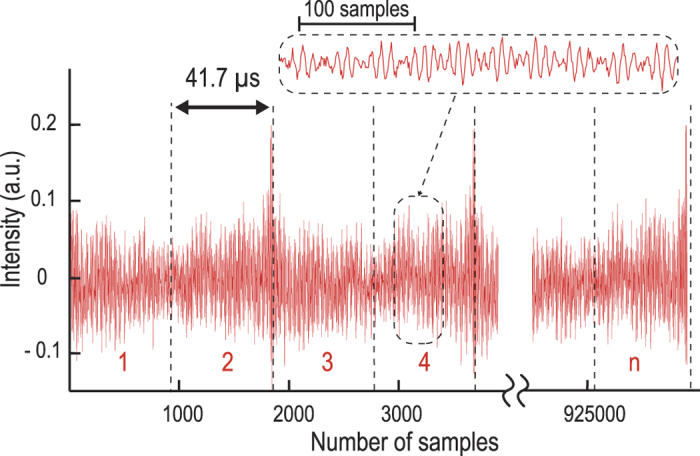
Continuous time-domain CARS interferograms of liquid toluene obtained by the rapid-scanning FT-CARS system. The CARS interferograms are continuously measured every 41.7 μs. The large peaks that appear every 83.3 μs are caused by the non-resonant background due to the zero time delay between the interferometer arms and are removed to avoid spectral distortions. The inset shows a zoom of the CARS interferogram.

**Figure 4:**
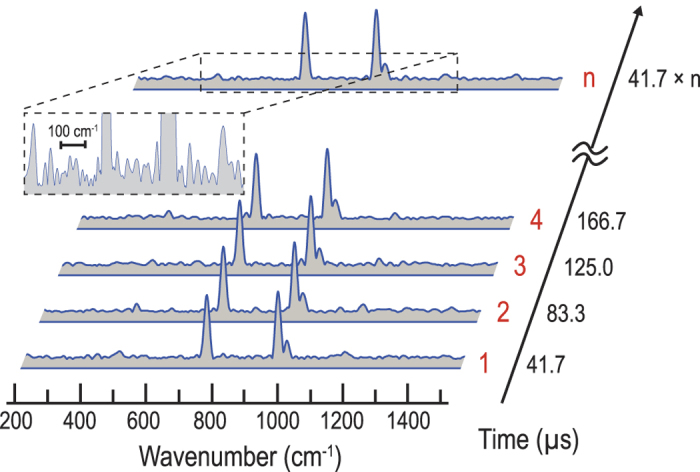
Continuous CARS spectra of toluene in the fingerprint region obtained by the rapid-scanning FT-CARS system. Each CARS spectrum is obtained by segmenting and Fourier-transforming each interferogram in the time-domain waveform in Fig. 3. Consequently, the CARS spectrum is acquired at an ultrashort scan period of 41.7 μs/spectrum, corresponding to a scan rate of 24,000 spectra/s.

**Figure 5:**
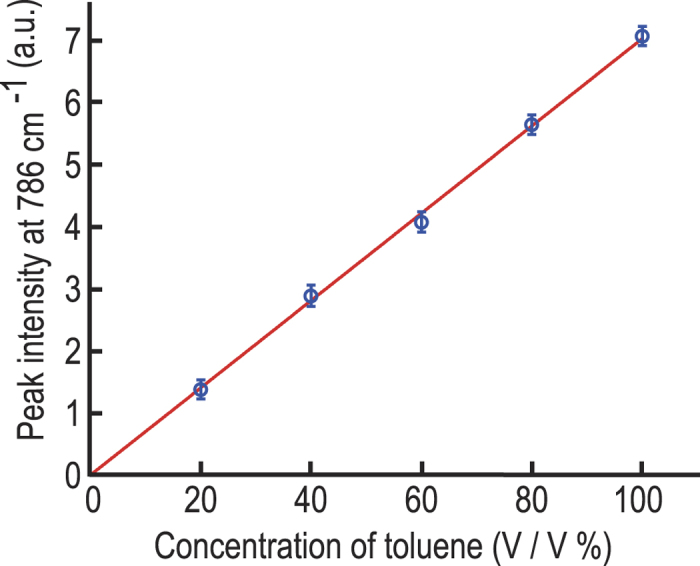
Dependence of the CARS signal on the concentration of toluene. The peak intensity of toluene at 786 cm^−1^ is proportional to the toluene concentration. Based on the noise level, the detection limit of our system on the toluene concentration is estimated to be 3%.

**Figure 6:**
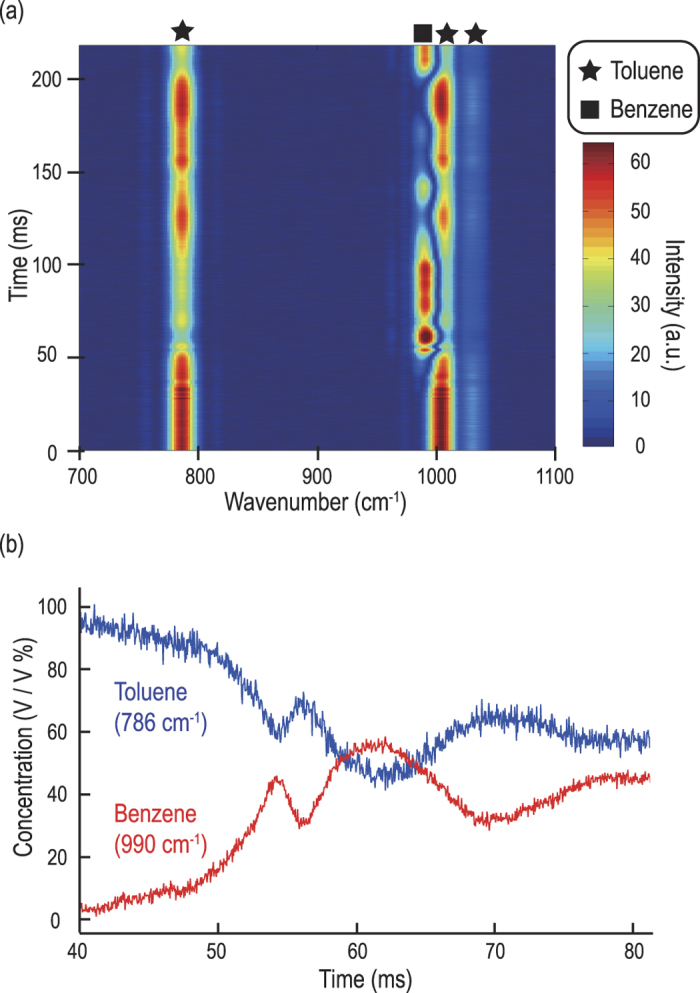
Mixing dynamics of toluene and benzene captured by the rapid-scanning FT-CARS system. (**a**) Evolution of the CARS spectrum during the mixing process of toluene and benzene. Here benzene is added to and mixed with toluene. In the beginning, only the CARS signal of toluene is present whereas at a later time, the CARS signatures of the two species are present, indicating that they are mixed. (**b**) Temporal variations in the concentration of toluene and benzene.
